# Negative Regulation of the IL-1 System by IL-1R2 and IL-1R8: Relevance in Pathophysiology and Disease

**DOI:** 10.3389/fimmu.2022.804641

**Published:** 2022-02-08

**Authors:** Domenico Supino, Luna Minute, Andrea Mariancini, Federica Riva, Elena Magrini, Cecilia Garlanda

**Affiliations:** ^1^ Department of Immunology and Inflammation, IRCCS Humanitas Research Hospital, Rozzano, Italy; ^2^ Department of Biomedical Science, Humanitas University, Pieve Emanuele, Italy; ^3^ Department of Veterinary Medicine, University of Milan, Milan, Italy

**Keywords:** inflammation, toll-like-receptors, negative regulation, innate immunity, interleukin 1

## Abstract

Interleukin-1 (IL-1) is a primary cytokine of innate immunity and inflammation. IL-1 belongs to a complex family including ligands with agonist activity, receptor antagonists, and an anti-inflammatory cytokine. The receptors for these ligands, the IL-1 Receptor (IL-1R) family, include signaling receptor complexes, decoy receptors, and negative regulators. Agonists and regulatory molecules co-evolved, suggesting the evolutionary relevance of a tight control of inflammatory responses, which ensures a balance between amplification of innate immunity and uncontrolled inflammation. IL-1 family members interact with innate immunity cells promoting innate immunity, as well as with innate and adaptive lymphoid cells, contributing to their differentiation and functional polarization and plasticity. Here we will review the properties of two key regulatory receptors of the IL-1 system, IL-1R2, the first decoy receptor identified, and IL-1R8, a pleiotropic regulator of different IL-1 family members and co-receptor for IL-37, the anti-inflammatory member of the IL-1 family. Their complex impact in pathology, ranging from infections and inflammatory responses, to cancer and neurologic disorders, as well as clinical implications and potential therapeutic exploitation will be presented.

## Introduction

The pro-inflammatory cytokine interleukin-1 (IL-1) was discovered during the 1970s and recognised for its functions in inflammation, in particular in fever, lymphocyte activation, and hematopoiesis (1). Gene cloning and molecular identification of IL-1- and IL-1-receptor-related molecules allowed the identification of the entire IL-1 family, which is now considered a “system” comprising evolutionarily conserved ligands and receptors. A new nomenclature of the family receptors has been recently proposed and reported here, followed by previously used names. The IL-1 system includes ligands endowed with agonist activity (IL-1α, IL-1β, IL-18, IL-33, IL-36α, IL-36β, and IL-36γ), receptor antagonists (IL-1Ra, IL-36Ra, and IL-38) and an anti-inflammatory cytokine (IL-37), and receptors acting as signalling molecules (IL-1R1, IL-1R4/ST2, IL-1R5/IL-18Rα, IL-1R6/IL-1Rrp2/IL-36R), accessory proteins (IL-1R3/IL-1RAcP, IL-1R7/IL-18Rβ), decoy or negative regulatory receptors (IL-1R2, IL-1R8/SIGIRR/TIR8). Finally, the system includes receptors which are still considered orphan or whose function is poorly defined (IL-1R9/TIGIRR-2, IL-1R10/TIGIRR-1).

After gene transcription and translation in response to inflammatory signals or tissue damage, ligands of the IL-1 family, in particular IL-1β and IL-18, remain in the cytoplasm as precursors, and are then cleaved intracellularly by the inflammasome and Caspase-1 (2), or processed extracellularly by proteases, such as neutrophil protease proteinase-3, elastase, matrix metalloprotease 9 and granzyme B, reaching their optimal biological activity. In contrast with other members of the family, IL-1α is constitutively expressed by several cell types, and can act as an alarmin also in its precursor form, when released upon tissue damage or exposed as an integral membrane protein (3–5).

IL-1-family receptors have a structure comprising a ligand-binding extracellular portion consisting of three Ig-like domains, and an intracellular TIR domain (originally an acronym for Toll/IL-1-resistance and now for Toll/IL-1R domain), which is essential for signaling *via* the MyD88 adaptor and shared by TLRs. Upon ligand-binding, the main receptor chain and the accessory protein chain assemble in a heterodimer and the TIR domains activate a phylogenetically conserved signaling cascade. The signaling pathway includes the TIR-containing adaptor molecule MyD88, downstream protein kinases (e.g. IL-1R associated kinases (IRAKs), and tumor necrosis factor receptor-associated factor 6 (TRAF6)) and leads to NF-κB translocation to the nucleus and activation of mitogen−activated protein kinases (MAPKs), such as p38, c−Jun N−terminal kinases (JNKs) and extracellular signal−regulated kinases (ERKs), resulting in amplification of innate immunity and inflammation (6).

Five signaling receptor complexes, constituted by a main receptor chain and an accessory receptor chain, are responsible of cell activation after the interaction with IL-1 family members: the IL-1 receptor (IL-1R1 and IL-1R3/IL-1RAcP) which binds IL-1α and IL-1β; the IL-33 receptor (IL-1R4/ST2 and IL-1R3/IL-1RAcP); the IL-18 receptor (IL-1R5/IL-18Rα and IL-1R7/IL-18Rβ); the IL-36 receptor (IL-1R6/IL-1Rrp2 and IL-1R3/IL-1RAcP) which binds IL-36α, β and γ; and the recently identified IL-37 receptor (IL-1R5/IL-18Rα and IL-1R8).

The IL-1 system is generally associated with inflammation and innate immunity. However, the members of this family, in particular IL-1, IL-33 and IL-18, are now known to play broader and complex roles, which include orienting innate immunity and inflammation in response to microbial or environmental challenges, and promoting differentiation and polarization of myeloid cells and innate or adaptive lymphoid cells.

Phylogenetic analysis showed that agonists, receptor antagonists, anti-inflammatory molecules and IL-1 receptor family members coevolved, since most of them (IL-1β, IL-1Ra, the IL-36 subgroup, IL-38 and IL-37, IL-18) are present in all vertebrates (7). This suggests the relevance in evolution of IL-1 system regulation, mediated by antagonists and anti-inflammatory cytokines, as well as by decoy or regulatory receptors. Among these, IL-1Ra and IL-36Ra are receptor antagonists that compete with the agonists IL-1 and IL-36 for the interaction with IL-1R1 and IL-1R6, respectively, thus reducing their activity (2), whereas IL-18BP is a soluble molecule that binds IL-18, preventing the interaction with its receptor (8). IL-1R2 lacks a signaling TIR domain and acts in a membrane or soluble form as a decoy receptor for IL-1 (9). IL-1R8, also known as TIR8 or SIGIRR, behaves as a negative regulator of the signal transduction by other members of the family, by interfering with the association of TIR-containing adaptor molecules to the receptor complex (10). In addition, in association with IL-1R5/IL-18Rα, IL-1R8 has been shown to act as co-receptor for the anti-inflammatory cytokine IL-37 (11), thus opening several new lines of research on the role of IL-1R8 in immunopathology.

Decoy receptors are also strategies of immune evasion adopted by pathogens. For instance, DNA viruses encode proteins homologous to mammal decoy receptors; in particular, Poxviruses express a soluble version of IL-1R (12). In addition, several bacteria (e.g. *Brucella melitensis*, *Escherichia coli*, *Salmonella enterica*, *Pseudomonas denitrificans* and *Pseudomonas aeruginosa*) have evolved TIR-containing proteins (Tcps) that dampen TIR-related pathways (13–16). These data suggest that genomic recombination events originated pathogens endowed with anti-inflammatory molecules from the host genome, which may favor infection and pathogen persistence.

Here we review the regulatory roles of IL-1 receptor family members, focusing on IL-1R2, the first “decoy” receptor identified, and IL-1R8, which being expressed by different cell types and acting as negative regulator of several IL-1 family members, as well as of TLRs, has pleiotropic functions in several pathophysiological contexts involving inflammation and innate and adaptive immune responses.

## The Decoy Receptor IL-1R2

### IL-1R2 Mode of Action

The *IL-1R2* gene is located in chromosome 2, in the locus including the IL-1R cluster, e.g. IL-33, IL-18, and IL-36 receptors. Like other IL-1R family members, IL-1R2 protein is composed of an extracellular portion containing three extensively glycosylated immunoglobulin (Ig)-like domains, and showing 28% amino-acid homology with IL-1R1 extracellular portion. But in contrast with the other members of the family, IL-1R2 lacks the characteristic intracellular TIR domain, that is replaced by a 29 amino acid-long tail. Due to this peculiarity, this receptor is unable to initiate signal transduction following the interaction with its ligands (9, 17).

**Figure 1:**
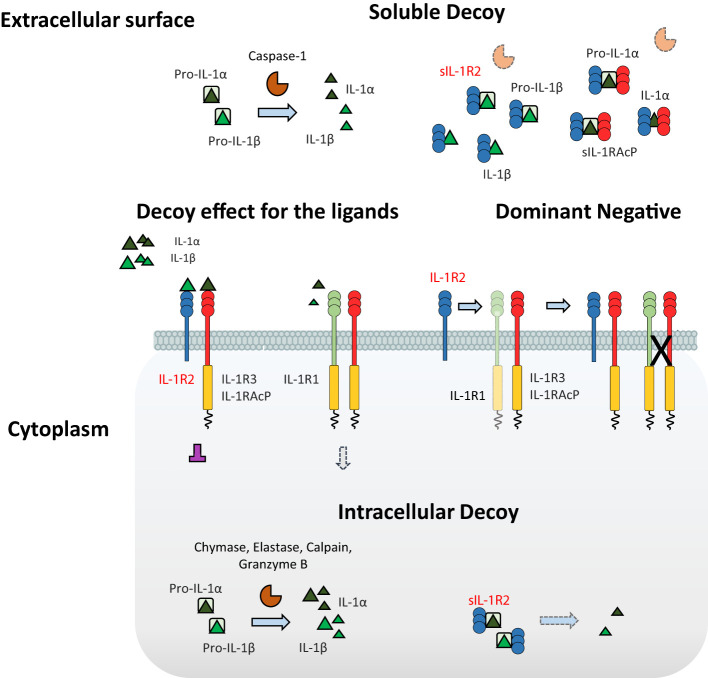
Negative regulation of IL-1-mediated pathways by IL-1R2. IL-1R2 differs from the other ILRs for the absence of the characteristic intracellular TIR domain, thus being incapable of signaling. IL-1R2 influences several mechanisms involved in the IL-1-mediated signaling cascade. IL-1R2 interacts with IL-1R3, acting as a dominant negative and impeding the formation of the IL-1R1//IL-1R3 signaling receptor complex; then, IL-1R2//IL-1R3 prevents the interaction between the ligands and the IL-1R1//IL-1R3 complex, by competitive binding to the pro-inflammatory cytokines IL-1α and IL-1β, thus acting as a decoy for the ligands. In addition, sIL-1R2 acts as a soluble decoy by binding IL-1α and IL-1β, as well as pro-IL-1β, blocking its enzymatic cleavage by caspase-1. The interaction of sIL-1R2 with the soluble form of IL-1R3 further increases the affinity for the ligands. Finally, in cytosol soluble form, IL-1R2 regulates the pro-inflammatory activity of IL-1α by preventing the enzymatic cleavage of pro-IL-1α, acting as an intracellular decoy.

IL-1R2 affects several steps along the IL-1-mediated signaling cascade (**Figure 1**). First, IL-1R2 acts as dominant negative molecule since it prevents the formation of IL-1R1//IL-1R3 complex by sequestering IL-1R3 (9, 18, 19). Then, IL-1R2//IL-1R3 competes with IL-1R1//IL-1R3 for the interaction with the ligands, since both receptor complexes recognize the pro-inflammatory cytokines IL-1α and IL-1β (20, 21). In addition, the enzymatic cleavage of IL-1R2 or alternative splicing generate a soluble form of the receptor (sIL-1R2) that exhibits anti-inflammatory activity by sequestering IL-1 (22–25). The enzymatic cleavage of IL-1R2 is mediated by the metalloproteinase ADAM17, which is activated by pro-inflammatory stimuli such as TNFα, LPS, leukotriene B4 and fMLF (26–28). sIL-1R2 is physiologically released into the bloodstream, where it binds IL-1α and IL-1β (18, 29), as well as pro-IL-1β preventing its enzymatic cleavage by caspase-1 (30). The interaction of sIL-1R2 with the soluble form of IL-1R3 (detectable at high circulating concentration, 300ng/ml) further increases the binding affinity for pro-IL-1β. In addition, cytosolic IL-1R2 interacts with pro-IL-1α preventing its enzymatic cleavage by calpain and other inflammatory proteases, thus tuning IL-1α-dependent sterile inflammation (31). This complex is abrogated by caspase-1 which cleaves IL-1R2, allowing cleavage and secretion of IL-1α and restoration of its activities. Low intracytoplasmic expression of IL-1R2 was described in vascular smooth muscle cell (VSMC) and activated macrophages and was considered implicated in necrosis-associate inflammation (31).

Recently, a cell-surface pro-form of IL-1α (csIL-1α) was identified in macrophages (32). IL-1R2 and glycosylphosphatidylinositol (GPI) were reported to anchor csIL-1α on the plasma membrane restraining its activation and release. IL-1R2-deficient Bone Marrow-Derived Macrophages (BMDMs) displayed low levels of csIL-1α, highlighting the contribution of IL-1R2 in tethering IL-1α. Moreover, IL-1α trafficking from the cytoplasm to the plasma membrane was specifically inhibited by the stimulation with IFNγ, suggesting that macrophage polarization plays a crucial role in the regulation of csIL-1α (32).

Collectively, these studies show that IL-1R2 may regulate IL-1 through different mechanisms, acting at the cell membrane level, intracellularly or as a soluble molecule.

### IL-1R2 Expression

IL-1R2 was first identified on neutrophils, monocytes, macrophages, dendritic cells (DCs) and B cells in both human and mice (26, 33). Polarization of myeloid cells strongly influences the expression of IL-1R2, emphasizing its relevance in immune response orientation. In particular, “M2” anti-inflammatory stimuli such as IL-4, IL-13, IL-10, IL-27, glucocorticoid hormones, prostaglandins and aspirin up-regulate IL-1R2 (9, 34–38). In contrast, stimulation with “M1” pro-inflammatory molecules (e.g. LPS, IFNγ and TNFα), chemoattractants and reactive oxygen intermediates leads to down-regulation of IL-1R2 (22, 28).

Regulation of IL-1R2 expression has been described in different cell types as a mechanism which counterbalances exacerbated inflammation in response to exogenous stimuli. For instance, up-regulation of IL-1R2 in microglial cells and brain endothelial cells attenuated central nervous system (CNS) inflammation in experimental models of IL-1β-induced-neurotoxicity (e.g. central administration of IL-1β, kainic acid administration, cerebral ischemia) (39–41). Human atherosclerotic vessels and monocytes/macrophages were reported to express low levels of IL-1R2 in hyperlipidemic patients (42). Moreover, IL-1R2 was down-regulated in THP-1 cells stimulated with acetylated low density (ac-LDL) and very low density (VLDL) lipoproteins, suggesting a mechanistic link between familial hyperlipidemia and susceptibility to IL-1β-mediated inflammation (42).

In the context of inflammation-dependent bone remodeling, IL-1R2 was poorly expressed by large osteoclasts, a cell population involved in exacerbation of bone loss in response to IL-1, compared to small osteoclasts (43). Similar observations have been reported in osteoarthritis (OA), a disease in which IL-1β contributes to joint inflammation and progressive tissue destruction. Human OA chondrocytes, synovial and epithelial cells express low levels of IL-1R2 on the cell membrane. However, sIL-1R2 significantly inhibited the pro-inflammatory activity of endogenous IL-1β, thus influencing proteoglycan biosynthesis, as well as nitric oxide (NO) and prostaglandin E2 (PGE2) production in immortalized cell lines and chondrocytes from OA patients (44), confirming its anti-inflammatory role.

Complex and sometimes conflicting results have been reported on IL-1R2 expression by lymphocytes. Regulatory T cells (Tregs) have been shown to express IL-1R2 following TCR stimulation (45). In the mouse, IL-1R2^+^ was expressed by a subset of activated Tregs which recirculate from thymus to tissues. By inhibiting IL-1β, this subset contributed to thymus-derived FOXP3^+^ Treg maturation (46). Ritvo et al. showed that IL-1R2 is expressed by a subset of FOXP3^+^ Follicular regulatory T (Tfr) cells and that it contributed in tuning IL-1β-dependent activation of Follicular helper T (Tfh) cells, as well as their proliferation and cytokine production, thus limiting the germinal center (GC) reaction. Flow cytometric analysis confirmed that Tfr cells of human lymphoid tissues express IL-1R2, which in contrast with previous studies, was not detected in Treg (47).

In association with IL-23, IL-1 promotes IL-17 production by human and murine T cells, contributing to Th17-related diseases (10). IL-1R2 was shown in a subset of TCR-stimulated IL-1R1^+^ CD4^+^ T cells, and to regulate Th17 functional activation by limiting IL-1β responsiveness. In this context, IL-1R2 expression is regulated by the NFAT/FOXP3 complex which binds to the IL-1R2 promoter (48). Since IL-1R2 may be expressed by both Th17 cells and Tregs, based on these results, IL-1R2 has been proposed to be involved in the plasticity of these cells, in particular in the trans-differentiation of Th17 into Treg and contributing to the resolution of inflammation (48, 49).

Taking advantage of single cell-RNA (scRNA) sequencing, it has been shown that tumor-infiltrating Tregs express high levels of IL-1R2 compared to other lymphocytes, in particular in breast, colorectal or non-small-cell lung cancers (50, 51). Conversely, low percentage of IL-1R2^+^ Treg cells was reported among circulating CD45RO^+^ lymphocytes from colorectal cancer patients (52). IL-1R2^+^ Treg clonality was investigated by combining scRNA-sequencing with TCR sequencing in an experimental model of skin graft, mimicking human papillomavirus (HPV)–associated epithelial hyperplasia (53). This inflammatory condition was associated with increased recruitment of non-antigen specific Tregs, which displayed two major functional states characterized by high expression of *Il1r2* or *Klrg1*, and associated with a tumor-infiltrating and a tissue-resident signature, respectively (53). Analysis of Treg heterogeneity by scRNA-sequencing revealed a subset of potent immunosuppressive cells governed by the transcription factor IRF-4 in non-small-cell lung cancer (NSCLC), which expressed high levels of IL-1R2 (54). Along the same line, in another scRNA-sequencing study of NSCLC, IL-1R2 was one of the most upregulated gene in a cluster of tumor antigen experienced Treg cells characterized by the expression of TNFRSF9^+^ (4-1BB) and was associated with poor prognosis (55). Collectively, these results suggest that IL-1R2 expression is associated with specific Treg cell clusters, representing differential maturation or activation states, developed in pathological conditions, in particular in cancer. However, the stimuli that promote IL-1R2 expression in tumor-infiltrating Treg cells and the function of IL-1R2 in this subset are still to be identified.

Finally, IL-1R2 was induced by IL-33 in Group 2 Innate lymphoid cells (ILC2s) and was associated with decreased *Il5* and *Il13* transcripts following IL-33 stimulation, suggesting it acts as an activation-induced negative regulatory feedback mechanism that decreases ILC2 responsiveness to IL-33 (56).

### IL-1R2 in Infections

**Figure 2:**
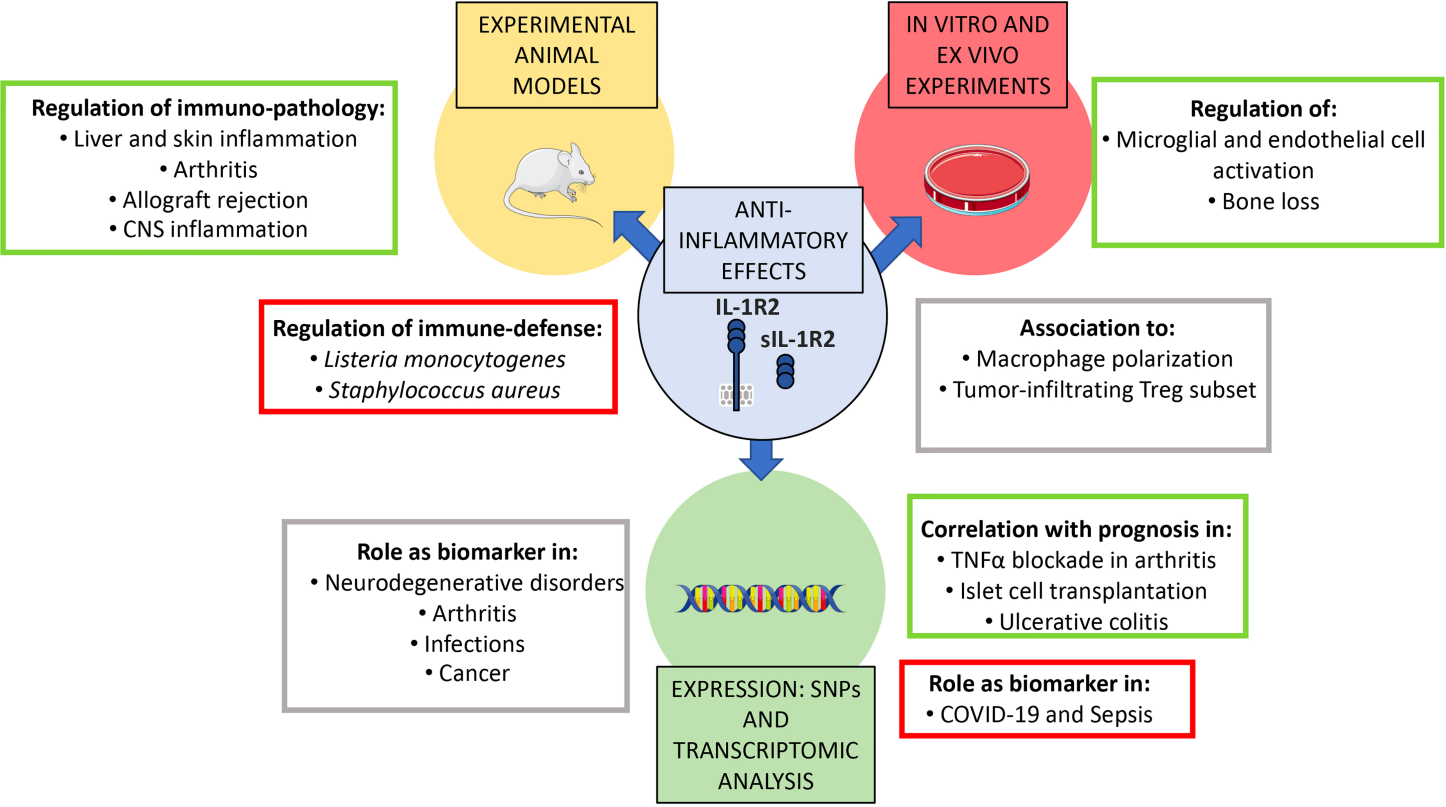
Roles of IL-1R2 in pathology. Experimental animal models, *in vitro* and *ex vivo* experiments, as well as scRNA-sequencing and transcriptomic analysis demonstrated the role of IL-1R2 as a key modulator and as a biomarker of acute and chronic inflammation in several pathological contexts. In particular, IL-1R2 plays a non-redundant role in regulating macrophage polarization, anti-microbial response and infections (such as sepsis and COVID-19), sterile inflammation (such as arthritis, liver, skin and CNS inflammation), neurodegenerative disorders and cancer. Full-length and sIL-1R2 have also been proposed as novel biomarkers for prognosis in infectious diseases, neurodegenerative disorders, rheumatoid arthritis, ulcerative colitis and cancer.

Even though inflammation is necessary to fight infections, deregulated immune reactions contribute to infection severity leading to tissue damage and spreading of pathogens from compartmentalized anatomical sites to circulation (57). Indeed, contrasting results emphasize the context-dependent role of IL-1β-mediated inflammation and regulation by IL-1R2 in different infective conditions, as described below (**Figure 2**).

In an experimental model of *Listeria monocytogenes* infection, higher frequency of bone marrow (BM) CD115^dim^IL-1R2^+^ monocytes was reported in lethal versus sub-lethal infections (58). In particular, up-regulation of IL-1R2 was associated with reduced production of IL-6 and ROS after stimulation with LPS, suggesting IL-1R2 contributes to the behavior of monocytes, which act as Trojan horses rather than bactericidal effector cells in this infection (58). In addition, *Staphylococcus aureus* was reported to evade from the immune surveillance by affecting the circulating levels of sIL-1R2 protein (59). Indeed, the virulence factor protein A triggered IL-1R2 shedding from monocytes and neutrophils through the activation of ADAM17. High levels of sIL-1R2 attenuated IL-1β-mediated inflammation and the anti-microbial response, including the production of IFNγ and TNFα (59). *Porphyromonas gingivalis* infection, which is associated with oral squamous cell carcinoma (OSCC), influenced myeloid polarization favoring the “M2”-like phenotype in macrophages and the upregulation of IL-1R2 and protumor molecules in cancer cells (60).

A scRNA-sequencing study of urinary-tract infection (UTI) patients identified IL-1R2 as one of the markers of a subset of CD14^+^, HLA-DR^low^ monocytes, which expand in different sepsis cohorts (61). In addition, IL-1R2^+^ cells were functionally exhausted as suggested by poor TNFα production upon stimulation with LPS. By mimicking sepsis-induced myelopoiesis, it was shown that IL-1R2^+^ monocytes originate from bone marrow mononuclear cells differentiated/activated by pathogen associated molecular patterns (PAMPs) (61). Along the same line, analysis of immune cells collected from COVID-19 patients indicated that “sepsis-associated” myeloid cells significantly increased in COVID-19 patients (62). As described in sepsis, HLA-DR^low^ IL-1R2^+^ cells showed impaired activation upon stimulation with LPS, suggesting that monocytes underwent function exhaustion as a consequence of the viral infection (62). Whether IL-1R2 is only a marker of monocyte dysfunction or is functionally implicated in this cell state, is still to be defined.

### IL-1R2 in Sterile Inflammation

Over the past decade, the biological role of IL-1R2 in the regulation of inflammation has been investigated by taking advantage of IL-1R2-deficient or overexpressing mice and experimental models of inflammatory diseases (**Figure 2**). Pioneering experiments demonstrated that transplantation of sIL-1R2-secreting keratinocytes ameliorated collagen-induced arthritis in mice (63). In agreement with this observation, increased susceptibility to arthritis was confirmed in *Il1r2* ^-/-^ mice, which was associated with increased production of inflammatory mediators such as IL-6, CXCL2, NOS2, and IL-1β by IL-1R2-deficient macrophages (64). The relevance of IL-1R2 in tuning inflammation was further highlighted in the K/BxN serum transfer arthritis model (65). Neutrophils express high levels of IL-1R2, but no significant difference in the effector functions of IL-1R2-deficient neutrophils was reported. However, neutrophils were shown to act in trans by releasing sIL-1R2, which in turn inhibited IL-1β-mediated activation of fibroblasts, thus regulating the expression of inflammatory molecules (e.g. IL-1β, IL-6, CXCL-1 and CXCL-2) in ankles. In contrast, IL-1R2-deficiency did not affect severity and mortality following acute administration of IL-1β or LPS, suggesting that IL-1R2 is primarily involved in regulating local inflammation (65). In support of this concept, IL-1R2 was described as a critical molecule in resolving liver inflammation (66). In particular, neutrophils up-regulated IL-1R2 in a liver injury model and contributed to protecting mice from hepatic deterioration, as confirmed by the adoptive transfer of this subset in the early stage of liver damage (66).

In experimental models of skin inflammation, constitutive expression of IL-1R2 by transgenic keratinocytes was associated with reduced production of granulocyte/macrophage colony-stimulating factor (GM-CSF) upon stimulation with IL-1β (67). Moreover, PMA-induced vascular permeability was reduced in IL-1R2 transgenic mice (67). In an experimental model of endometriosis, sIL-1R2 inhibited the expression of adhesion molecules (e.g. αv and β3 integrins), vascularization and tissue growth of transplanted human endometrium in nude mice (68).

Overexpression of IL-1R2 in the heart ameliorated cardiac allograft survival by controlling the production of pro-inflammatory mediators (e.g. IL-1β, TNFα, prostaglandin E2 synthase, cyclooxygenase, and CCL1) and Th17 polarization (69, 70). Recently, the transcription factor PAX6 was demonstrated to regulate IL-1R2 in cardiac fibroblasts. PAX6 promoted the expression of the anti-fibrotic factors IL-1R2 and CXCL10 and downregulated the pro-fibrotic molecule TGFβ1. In contrast, angiotensin II repressed PAX6/IL-1R2 thus triggering differentiation of fibroblast and cardiac fibrosis (71).

### IL-1R2 in Human Cancer

IL-1 is involved in carcinogenesis and metastasis, contributing to oncogene-driven and microenvironment-driven cancer related inflammation (72). Several studies investigated IL-1R2 expression in cancer cells or in the tumor microenvironment, to elucidate whether the IL-1R2 could be involved in tuning IL-1-dependent cancer-related inflammation. Data generated until now show that IL-1R2 is generally up-regulated in tumor tissue (**Figure 2**). In particular, *IL1R2* gene was up-regulated in pancreatic ductal adenocarcinoma (PDAC) and was proposed to protect cancer cells from apoptosis induced by IL-1 (73). In addition, *IL1R2* was included in a signature consisting of 9 genes that predicted tumor stages and survival of PDAC patients (74). In acute myeloid leukemia (AML), *IL1R2* gene expression was associated with bad prognosis (75), in ovarian cancer, *IL1R2* was upregulated in recurrent compared to primary cancer (76) and in prostate the molecule was expressed in prostatic cancer cells but not in normal cells (77). In gastric cancer, high expression of IL-1R2 in tumor tissue and increased levels of sIL-R2 in plasma were associated with poor prognosis (78). Moreover, gastric cancer ascites scRNA-seq analysis suggested that IL-1R2-expressing tumor cells contributed in tuning tumor-associated macrophage-dependent IL-1β-mediated inflammation (79).

In addition, as reported above, IL-1R2 recently emerged as a tumor-infiltrating Treg associated marker in scRNA sequencing studies, in breast, colorectal or non-small-cell lung cancers, together with several immune-checkpoints (50, 51). However, further studies are needed to elucidate the functional role of IL-1R2 in tumor-infiltrating Treg cells and other immune cells.

Collectively, these results suggest that IL-1R2 is induced in cancer cells, often correlating with bad prognosis, and in tumor infiltrating leukocytes. However, genetic analyses in mouse or humans formally proving the actual role of IL-1R2 in cancer are still lacking. In particular, functional studies are needed to address whether IL-1R2 is part of a signature associated with immunosuppression as suggested by data on Tregs, or whether its induction reflects cancer-related inflammation, thus explaining the link with poor prognosis.

### IL-1R2 as a Potential Prognostic Biomarker

The soluble form of IL-1R2 is released from the cells in inflammatory conditions. For this reason, sIL-1R2 has been investigated as a potential biomarker of inflammatory disease. Results collected over the last years show that several inflammatory diseases are associated with increased release of sIL-1R2 (**Figure 2**). In particular, high concentration of circulating sIL-1R2 was reported in necrotizing enterocolitis (80), acute respiratory distress syndrome (81), acute meningococcal infection (82), Dengue (83) and sepsis (84). In these conditions, sIL-1R2 often reflected the severity of the condition.

In other contexts, such as rheumatoid arthritis, sIL-1R2 concentration negatively correlated with the severity of the condition, indicating that endogenous sIL-1R2 may constitute a natural anti-inflammatory factor in chronic polyarthritis (85). In agreement, monocyte production of sIL-1R2 correlated with favorable prognosis and efficacy of TNFα blockade with Etanercept in arthritis (86). Similarly, in multiple sclerosis, sIL-1R2 concentration increased in cerebrospinal fluid in response to steroid therapy, suggesting a beneficial effect of the molecule (87). Finally, islet transplantation outcome and insulin independency positively correlated with IL-1R2 expression (88).

At the transcriptional level, *IL1R2* was part of a signature related to myeloid cell activation which was highly expressed in non-responder Kawasaki patients following immunoglobulin infusion (89), and of a signature associated with immune infiltrate in acute myocardial infarction (90). Finally, transcriptional and protein analysis showed that IL-1R2 was a favorable prognostic marker in ulcerative colitis, being up-regulated in intestinal mucosal cells from ulcerative colitis patients in remission phase (91).

Collectively, these studies indicate that IL-1R2 is induced in several conditions, but that the link with prognosis or severity of the disease is variable, possibly depending on the underlying pathogenetic mechanisms, the cell type involved and the mechanisms of induction. For these reasons, the development of IL-1R2 as biomarker or prognostic tool seems unlikely.

## IL-1R8 (TIR8/SIGIRR)

### IL-1R8 Mode of Action

**Figure 3:**
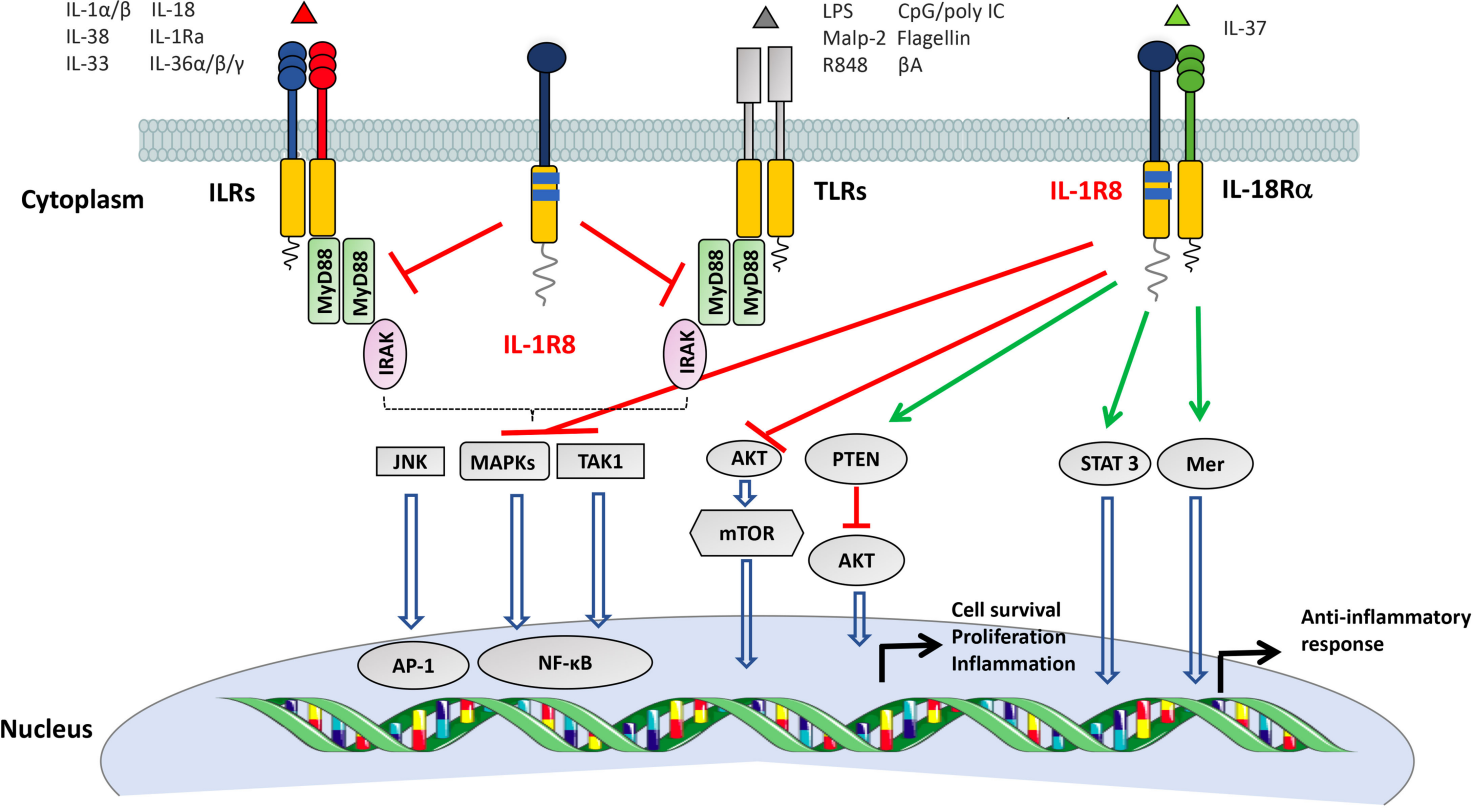
Mechanisms of negative regulation mediated by IL-1R8. IL-1R8 is characterized by a single extracellular Ig domain, a transmembrane domain, an intracellular TIR domain and an unusually long tail of 95 residues. The IL-1R8 TIR domain differs from the TIR domains of other ILRs for the substitution of two conserved residues (Ser447 and Tyr536 with Cys222 and Leu305), suggesting unconventional signaling. IL-1R8 inhibits the signalling pathways downstream ILRs and TLRs by competing with the recruitment of MyD88 and IRAK at the TIR domain, thus dampening the signaling pathways involved in NF-κB and JNK activation. In addition, in T, NK and epithelial cells, IL-1R8 negatively regulates the activation of the mTOR pathway. IL-1R8, together with IL-1R5, is part of the signaling receptor of the anti-inflammatory molecule IL-37. The IL-37//IL-1R5//IL-1R8 tripartite complex inhibits MAPK, NF-κB, mTOR, TAK1 and Fyn, and activates STAT3, Mer, PTEN and p62(dok) signalling, thus leading to an anti-inflammatory pathway.

IL-1R8 is a well conserved gene among vertebrates, including fish, located on human chromosome 11 and on mouse chromosome 7. The protein differs from other members of the family since IL-1R8 has a single Ig domain in the extracellular region which is N- and O-glycosylated, an unconventional intracellular TIR domain with two amino-acid substitutions in Ser447 and Tyr536, replaced by Cys222 and Leu305, influencing IL-1R8 signalling activity, and a long tail of 95 residues (**Figure 3**).

IL-1R8 is expressed by several cell types, in particular epithelial cells of the liver and kidney, and in lymphoid organs. Among leukocytes, IL-1R8 is highly expressed by DC, NK cells and T lymphocytes, and it is also expressed in platelets (92–95). In general, inflammatory conditions, such as treatment with LPS, are associated with downregulation of IL-1R8 expression (95–99) through the inhibition of SP1 binding on IL-1R8 promoter (96, 100).

Different IL-1R8 isoforms have been described, but their function is unknown. A longer isoform called IL-1R8L1 was characterized in tumor epithelial cell lines, in a neuroblastoma cell line, in leukemic cell lines, and in human healthy tissues (101).

The first functional roles described for IL-1R8 concern the inhibition of the signalling pathways downstream ILRs and TLRs (e.g. IL-1R1, IL-1R5/IL-18Rα, IL-1R4/ST2, TLR1, TLR2, TLR4, TLR7, TLR9, TLR3), leading to the reduction of NF-κB and JNK activation (94, 95, 102–107), (**Figure 3**). The molecular mechanism proposed indicates that IL-1R8 is recruited to the ligand-receptor complex, and the BB-loop structure of IL-1R8 TIR domain inhibits the dimerization of MyD88 (92, 93, 102, 104, 108, 109), or causes retention of the Myddosome complex on receptors without driving the pro-inflammatory cascade (110). In addition, the IL-1R8 extracellular domain has been shown to inhibit the interaction between IL-1R1 and IL-1R3 (104). In the case of TLR3 signalling, IL-1R8 blocked TRAM homodimerization and TLR4-TRAM and TRIF-TRAM interactions (110–112). IL-1R8 is also involved in the regulation of the mTOR pathway in lymphoid and not lymphoid cells (e.g. Th17, NK cells and intestinal epithelium) (94, 113, 114).

In addition, IL-1R8 is part with IL-1R5 of the signalling receptor of IL-37, an anti-inflammatory molecule of the IL-1 family, inducing an immunosuppressive pathway, inhibiting MAPK, NF-κB, mTOR, TAK1 and Fyn, and activating STAT3, Mer, PTEN and p62(dok) signaling (11, 115) (**Figure 3**). Upon ligation, IL-37 induced activation of glycogen synthase kinase 3β which promoted IL-1R8 phosphorylation, internalization, and degradation by the ubiquitin-proteasome system (116). Several studies showed that IL-1R8 is necessary for the anti-inflammatory potential of IL-37 in different pathologic conditions, including LPS-induced endotoxemia, *Aspergillus fumigatus* pulmonary infection (11, 117), allergic responses (118), neuroinflammatory diseases, such as multiple sclerosis (119) or spinal cord injury (120), and DSS-induced colitis (121). Further, IL-37 inhibited β-glucan-dependent trained innate immunity, an innate immune memory program induced in monocytes/macrophages by exposure to pathogens or vaccines, associated with protection against infections. In this context, IL-1R8 was required for the inhibitory role of IL-37 in the production of inflammatory mediators by monocytes (122). Finally, IL-37 alleviated endothelial cell apoptosis and inflammation *via* IL-1R8, by inhibiting ERK and NF-κB activation (123).

Activation of the IL-1R5//IL-1R8 receptor complex by IL-37 was also involved in tuning of mTOR signaling and activation of STAT6 and Foxo transcription factor family, with effects on metabolism, insulin response and glucose tolerance (11, 124). IL-1R8 was finally necessary for the activity of IL-37 in muscle cells, where it orchestrated the AMPK pathway and improved exercise performance and fatigue tolerance (125).

The regulatory role of IL-1R8 is conserved in evolution: for instance, in zebrafish IL-1R8 sequesters TRIF competing with its recruitment at the TLR3/TLR22 receptor complex, thus contributing to the maintenance of liver homeostasis under inflammatory conditions (126). In veterinary medicine, several reports show the expression and relevance of IL-1R8 in inflammatory and infectious conditions, including infection by porcine circovirus 2 in pigs (127) and *Staphylococcus aureus* mastitis in goat (128), inflammation in forestomach wall and mammary cells of ruminants (129–131), or intestinal epithelial cells and APCs from Peyer’s patches in pigs (132–134).

### IL-1R8 in Infections

**Figure 4:**
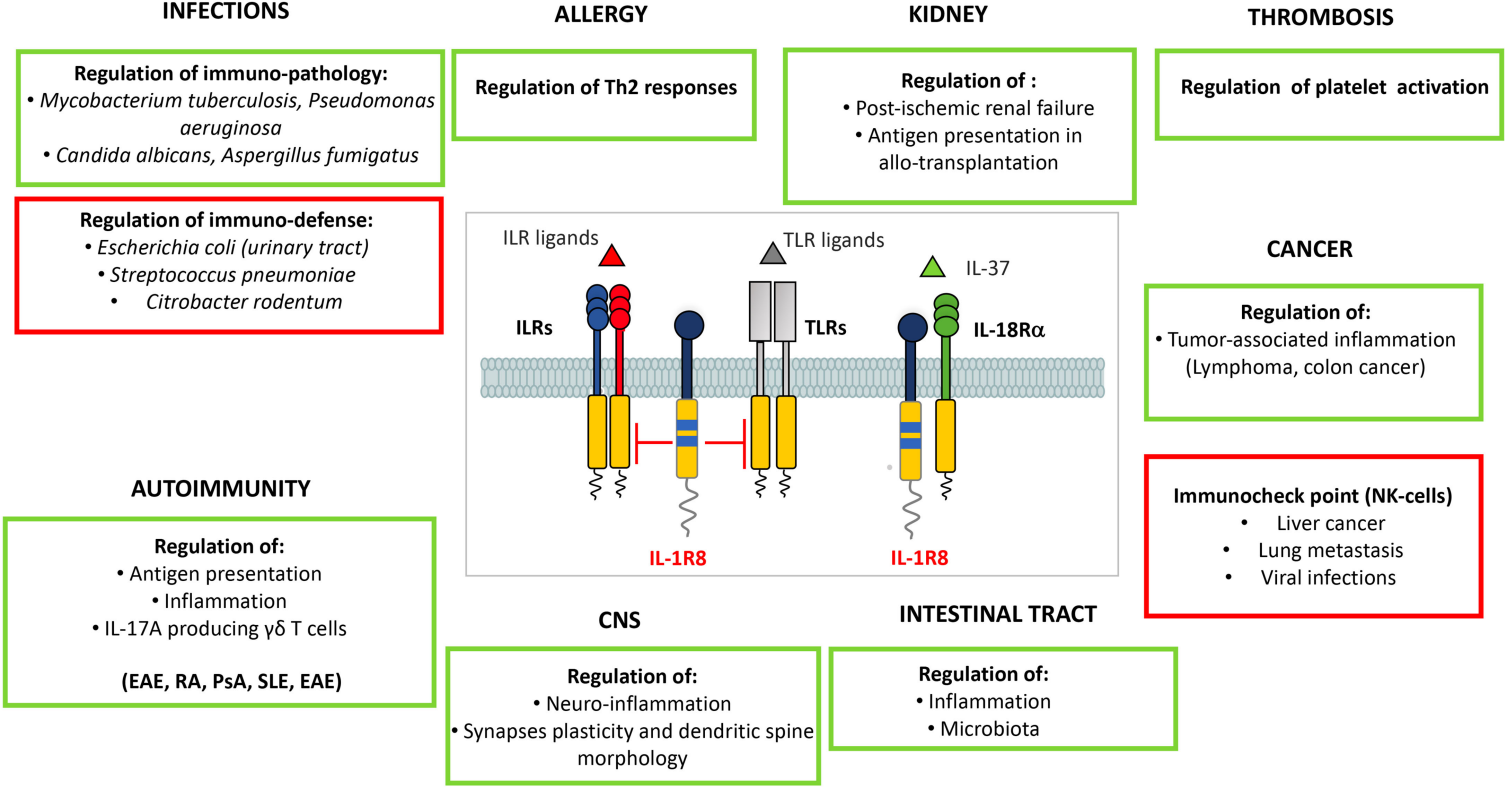
Roles of IL-1R8 in pathology. IL-1R8 fine tunes innate and adaptive immune responses in different pathological conditions, thus acting as a key modulator of inflammation. IL-1R8 plays a non-redundant role in bacterial and fungal infections, allergy, autoimmune diseases, renal inflammation, thrombosis, neuro-inflammation and neuronal plasticity, intestinal inflammation, and cancer (colorectal cancer, breast cancer, prostate cancer and CLL). Recently, IL-1R8 has emerged as a novel checkpoint molecule in NK cells. In particular, IL-1R8 modulates maturation and activation of murine and human NK cells, thus regulating their antiviral and antitumor potential. In infections, IL-1R8 plays a dual role: in green, the conditions in which IL-1R8 has a protective role by preventing immunopathology; in red, the specific infections in which by negatively tuning innate responses, IL-1R8 prevents the development of effective anti-microbial resistance. Similarly, in cancer, IL-1R8 tunes cancer-related inflammation in specific tumors (in green), or acts as a checkpoint for NK cells, restraining their anti-tumor and anti-metastatic (and anti-viral) potential.

IL-1R8 plays dual roles in different infections. Depending on the type of infection, IL-1R8 deficient mice developed more severe inflammation and tissue damage in several models of experimental infections, or on the other hand showed increased protective innate immune responses leading to higher resistance to the infection (**Table 1** and **Figure 4**). In a model of *Mycobacterium tuberculosis* infection, IL-1R8 deficient animals presented an increased mortality mainly due to an exaggerated inflammatory response with enhanced leukocyte infiltration in lung and higher levels of proinflammatory cytokines. The phenotype was rescued by the preventive administration of IL-1 and TNFα blocking antibodies (135). A genome wide association study aimed at identifying genetic variants associated with resistance to tuberculosis in bovine, an infection representing a risk to public health, identified IL-1R8 among genes associated with resistance to the infection (154). In line with these results, a strong association between 3 *IL1R8* SNPs (rs10902158, rs7105848, rs7111432) and tuberculosis infections was described in a study involving more than 600 patients and negative controls (155).

**Table 1:** Pathophysiological roles of IL-1R8 in disease.

Pathological context	Disease*	Role of IL-1R8	Modulated target**	Selected ref.
**Infection**	*Mycobacterium tuberculosis*	Prevention of liver necrosis, IL-1β/TNF mediated inflammation	IL-1R	(135)
	*Pseudomonas aeruginosa* (lung infection)	Prevention of high bacterial load and excessive inflammation	IL-1R	(98)
	*Candida albicans/* *Aspergillus fumigatus*	Prevention of Th17 response and pathogen dissemination	IL-1R	(136)
	*Escherichia coli* (pyelonephritis)	Susceptibility to renal dysfunction	TLR4	(137)
	*Streptococcus pneumoniae*	Susceptibility to mortality induced by pneumonia and sepsis	Unknown	(138)
	*Citrobacter rodentium*	Prevention of commensal bacteria loss and gut inflammation	IL-1R	(139)
	Human Immunodeficiency Virus (HIV)	Regulation of inflammation by IL-37 in HIV infected cells	IL-37	(140)
**Autoimmunity**	Lupus Nephritis/Systemic Lupus Erythematosus (SLE)	Prevention of autoantigen presentation and lupus autoantibodies production/Control of Th17 response	TLRs (TLR7)	(141)
	Rheumatoid Arthritis (RA)	Control of activation of myeloid and synovial cells	IL-1R TLRs	(112)
	Psoriatic Arthritis (PsA)	Prevention of IL-17A γδ T cell –mediated inflammation and IL-36	IL-1R and IL-36R	(142, 143)
	Multiple Sclerosis (MS)	Control of Th17 polarization, leukocyte infiltration in the brain and spinal cord	IL-1R	(113)
	Myasthenia Gravis (MG)	Control of Th and B cells proliferation and autoantibody secretion	IL-37	(144)
	Graft rejection	Control of donor antigen presentation, Th1 and Th17 responses	IL-1R	(145, 146)
**Allergy**	Hyperallergic pulmonary inflammation	Control of Th2 responses and prevention of severe disease	IL-33R; IL-37	(106, 118)
	House dust mite (HDM) asthma	Stimulation of Th2 responses, eosinophilic inflammation, mucus and HDM-specific IgG1 production	TLR-4	(147)
**Thrombosis**		Prevention of platelet and neutrophil-platelet aggregation	TLRs/IL-1R/IL-18R	(95)
**Neurological Dysfunctions**		Regulation of neuron synapse morphology, plasticity and functions. Regulation of hyppocampal function	IL-1R/TLR4/TLR2	(107, 148)
**Colitis**		Modulation of gut microflora and prevention of intestinal inflammation	TLRs	(103, 149)
**Cancer**	Colitis-associated cancer	Prevention of intestinal inflammation-associated cancer	TLRs	(114, 149, 150)
	Breast Cancer	Negative regulation of a protective tumor immune infiltrate	Unknown	(151)
	Hepatocellular carcinoma/Sarcoma lung metastasis/Colon Cancer metastasis	Immunocheckpoint in NK cells	IL-18R	(94)
	Chronic Lymphocytic leukemia/Diffuse large B-cell lymphoma	Prevention of monoclonal B cell expansion	Unknown	(152, 153)

*Selected.

**Demonstrated or proposed.

In *Pseudomonas aeruginosa* lung infection IL-1R8 deficient mice showed higher mortality, bacterial load and systemic and local levels of cytokines (IFNγ, IL-1β, TNFα, IL-6) compared to wild type mice. The phenotype was reverted by IL-1R1-deficiency, demonstrating that IL-1R8 prevented *P. aeruginosa* associated inflammation by negatively regulating IL-1R1, the major signaling pathway involved in the pathogenesis of this infection (98). In a model of *E. coli* pneumonia, the pro-resolving mediator 15-epi-lipoxin A_4_ induced the expression of A20 and IL-1R8 through a lipoxin A_4_ receptor/formyl peptide receptor 2 dependent mechanism, dampening lung inflammation and promoting pathogen clearance (156).

In fungal infections by *Candida albicans* and *Aspergillus fumigatus*, IL-1R8 deficient mice showed an increased susceptibility in terms of pathogen dissemination in tissues, mortality, and increased Th17 cell activation mediated by IL-1 signaling (136). Furthermore, as stated above, IL-1R8 was essential for the anti-inflammatory role of IL-37 in pulmonary aspergillosis.

On the other hand, in a model of *E. coli* pyelonephritis, the stronger inflammatory response of kidney epithelial cells to bacteria and PAMPs (LPS) protected IL-1R8 deficient mice from renal dysfunction, thanks to an increased recruitment of neutrophils in the early phase of infection (137). Along the same line, IL-1R8 deficiency protected mice from mortality in *Streptococcus pneumoniae* pneumonia and sepsis, and was associated with reduced bacterial load and dissemination (138).

IL-1R8 is highly expressed in gut epithelium and this has been linked to tuning of TLR reactivity against commensal bacteria; IL-1R8-deficiency in mice infected with *Citrobacter rodentium* was associated with exaggerated IL-1R1 signaling-dependent gut inflammation, causing a severe loss of commensal bacteria and facilitated secondary infection by *Salmonella typhimurium* (139). Along the same line, some probiotic bacteria used in the treatment of gut infections and diseases, beneficially regulated host immune responses by modulating TLR negative regulators, including IL-1R8 (157, 158).

An *in vitro* study demonstrated that IL-1R8 acts as a negative regulator of the immune response to *Chlamydia trachomatis*, by reducing the expression of IL-8 in infected epithelial cells (159).

In HIV infection, a significant correlation between TLR4 and IL-1R8 gene expression in brain and HIV-associated neurodegeneration was observed (160). Moreover, the IL-37/IL-1R8 axis was impaired in HIV infected subjects and associated with increased inflammation and viral replication, thus suggesting the therapeutic potential of the IL-37/IL-1R8 axis in HIV infection (140).

Thus, IL-1R8 emerges as a tuner of innate and inflammatory responses, and depending on specific infections, its role results in protection from immunopathology or limitation of protective immune responses against the pathogen.

### IL-1R8 in Sterile Inflammation: Autoimmunity, Graft Rejection and Allergy

IL-1R8 is involved in the regulation of TLR-dependent sterile inflammation associated with autoimmunity, in several models (**Table 1** and **Figure 4**). In C57BL/6-lpr/lpr mice, that develop a progressive lymphoproliferative syndrome followed by severe autoimmune disease and lupus nephritis, IL-1R8 deficiency was associated with higher activation of DCs and expression of IL-6, IFNβ, TNF, IL-12, and B cell survival factors Baff/BlyS and Bcl-2, as well as production of lupus autoantibodies (141). In the hydrocarbon oil-induced systemic lupus erythematosus (SLE) murine model, IL-1R8-deficiency was associated with unleashed TLR-7-mediated activation of DCs and consequent more severe autoimmune tissue injury (161). In SLE patients, the percentage of circulating IL-1R8^+^CD4^+^ cells inversely correlated with SLE severity and nephritis biomarkers concentration (162), and with the percentage of Th17 circulating cells, which proportionally increase with SLE severity (163).

In two different rheumatoid arthritis (RA) mouse models, IL-1R8-deficiency was associated with overactivation of myeloid and synovial cells, leading to a more severe disease in terms of clinical score or joint cellular infiltration (112). Based on the high expression of the IL-37//IL-1R5//IL-1R8 complex in CD4^+^ cells of RA patients, IL-37 has been proposed as a promising therapeutic target in RA (164, 165).

In psoriatic arthritis (PsA), a gene expression profile of PBMCs from patients and healthy controls showed that negative regulators of innate responses, including IL-1R8, are the genes that undergo the greatest reduction in expression (166). In line with these data, IL-1R8-deficient mice developed severe psoriatic inflammation in both chemical and cytokine-induced psoriasis mouse models, compared to wild type mice. These models depend on high IL-17A-expressing γδT cells, whose activity was suppressed by IL-1R8 (142). In addition, IL-1R8 negatively regulated IL-36-dependent psoriatic inflammation in humans and mice, acting in particular in dendritic cells and keratinocytes and modulating neutrophil chemo-attractants (143). The deficiency of IL-1R8 resulted in enhanced Th17 cell polarization *in vivo* in a multiple sclerosis (MS) mouse model (experimental autoimmune encephalomyelitis, EAE) and increased disease severity, characterized by higher leukocyte activation and infiltration in the brain and spinal cord (113). In this model, IL-1R8 was involved in the regulation of Th17 cell differentiation, expansion and functions, due to its inhibitory effects on IL-1 signaling leading to JNK and mTOR activation (113).

Recombinant IL-37 has been proposed as a novel therapeutic strategy for MS, since patients, despite the low production of endogenous IL-37, still present the receptor complex IL-1R5//IL-1R8 on their PBMCs and brain lesions (119). Along the same line, patients affected by myasthenia gravis (MG) presented lower IL-37 serum levels compared with healthy controls, which were associated with high follicular T helper and B cell numbers. Both populations express high levels of IL-1R8 and their stimulation with IL-37 results in reduced proliferation, cytokine production and secretion of autoantibodies, suggesting its therapeutic potential in MG (144).

In a model of kidney allotransplantation, IL-1R8-deficiency was associated with graft rejection, in contrast with IL-1R8-competent grafts which were spontaneously accepted. In this context, a major role was played by graft-resident DCs, which, when deficient of IL-1R8, exerted improved donor antigen presentation and stimulated the production of higher amounts of IFNγ by allogenic T cells (145). IL-1R8 overexpressing DCs also contributed to prolonged survival of allografts in an islet transplantation mouse model, by inducing weak systemic Th1 and Th17 responses that were counterbalanced by a strong Treg-mediated immunoregulation, leading to allografts survival (146).

The involvement of IL-1R8 in allergy appears at the moment controversial. Bulek K. et al. originally showed that IL-1R8-deficiency is associated with more severe hyperallergic pulmonary inflammation and that IL-1R8 is involved in T cell-mediated type 2 response by negatively regulating the IL-33/ST2 complex (106). In a mouse model of acute asthma, intranasal administration of rIL-37 ablated allergic airway inflammation, cytokine production, mucus hyperproduction and airway hyper-responsiveness, and these benefits were completely lost in IL-1R8 or IL-18Rα deficient mice (118). In contrast with these results, in a house dust mite (HDM) asthma model, which relies on the activation of TLR4 on epithelial cells and subsequent exacerbated Th2 specific response, IL-1R8-deficiency was associated with decreased production of Th2 cytokines in lung and draining lymph nodes, reduced eosinophilic inflammation, mucus production by goblet cells, HDM-specific IgG1 and airway hyperreactivity compared with wild type mice. The mechanism proposed was the up-regulation upon HDM sensitization of IL-1F5, a putative IL-1R8 ligand, an IL-4 inducer (147). Finally, a human genetic study based on exome sequencing on a cohort of Japanese patients with asthma excluded any association with IL-1R8 alleles or haplotypes (167).

Collectively, these studies in mouse underline the involvement of IL-1R8 in tuning inflammatory and immune responses activated in sterile conditions through engagement of TLRs or IL-1R family members. Even if fragmentary, evidence in human suggests the conservation in evolution of the regulatory functions of this molecule.

### IL-1R8 in Platelet Activation and Thromboembolism

IL-1R8 is expressed on different blood leukocytes, but platelets show the highest level of expression, both in humans and mice. High levels were also observed in megakaryocytes of both species (95). IL-1R8 deficiency was associated with platelet hyper-activation in basal conditions, increased platelet aggregation after prothrombotic stimulation and increased neutrophil-platelet aggregation induced by LPS, IL-1β and IL-18 *in vitro*. Indeed, platelets also express TLRs and IL-1 family members and IL-1R8 can regulate their signaling (95). In a model of ADP-induced pulmonary thromboembolism, IL-1R8 deficient mice were more susceptible mainly due to deregulated IL-1 signaling (95). Platelets from patients affected by SIRS/sepsis showed reduced IL-1R8 surface expression compared to platelets from healthy donors, reflecting the severity of the disease. Interestingly, expression of IL-1R8 in microvesicles released from platelets *in vitro* or found in plasma of sepsis patients suggests that IL-1R8 may be rapidly shed by the release of microvesicles in inflammatory conditions, contributing to platelet dysfunction observed in this inflammatory condition (95).

### IL-1R8 in the Central Nervous System

IL-1R8 is expressed in different types of cells in the brain such as neurons, astrocytes, and microglia. IL-1R8 deficient mice demonstrated impaired CNS development, leading to altered hippocampal capacity: difficulties in novel objective recognition, spatial reference memory and long-term potentiation (148) (**Table 1** and **Figure 4**). Neurological dysfunctions were associated to increased activation of IRAK1, JNK and NF-κB *via* IL-1R1 and TLR4 signaling after binding respectively to IL-1α and HMGB1 (148). Moreover, IL-1R8-deficiency and the consequent hyperactivation of the IL-1R pathway affected neuron synapse morphology, plasticity and function (107). IL-1R8-deficient hippocampal neurons displayed an increased number of immature, thin spines and a decreased number of mature, mushroom spines, along with a significant reduction of spine width, and reduced amplitude of miniature excitatory postsynaptic currents. The phenotype of IL-1R8-deficient neurons was associated with IL-1R1-driven hyperactivation of the PI3K/AKT/mTOR pathway, and increased expression of methyl-CpG-binding protein 2 (MeCP2), a synaptopathy protein involved in neurological diseases, such as Rett syndrome and MeCP2 duplication syndrome (168). Deficiency of IL-1R1 or treatment with IL-1Ra (Anakinra) normalized MeCP2 expression and cognitive deficits in IL-1R8-deficient mice, demonstrating that IL-1R8 fine tunes IL-1R1 signalling, is involved in synaptopathies and is required for correct long-term potentiation (107). In line with these results, the treatment with Anakinra of patients with cryopyrin-associated periodic syndrome (CAPS), in addition to reduce signs and symptoms of IL-1-dependent inflammation, reversed mental defects in patients (169). A further evidence of the relevance of IL-1R8 in the brain is provided by genetic studies on schizophrenia, which identified *SIGIRR* as one of the genes associated with genetic alterations in this psychiatric condition (170).

Finally, IL-1R8 also regulated β-amyloid peptide-dependent TLR2 activation in microglial cells and the release of the pro-inflammatory cytokines TNFα and IL-6 (171).

### IL-1R8 in Cancer

#### Intestinal Inflammation and Cancer

IL-1R8 plays an important role in gut homeostasis, intestinal inflammation and tumorigenesis (**Table 1** and **Figure 4**). In healthy mice, IL- 1R8 was shown to modulate gut microflora-mediated activation of ILRs and TLRs, which regulated the proliferation and survival of intestinal epithelial cells in colonic crypts. IL-1R8-deficient mice showed constitutive activation of NF-κB and JNK and increased expression of Cyclin D1 and Bcl-xL (149). The phenotype has not been confirmed by all the studies performed in healthy mice, probably due to animal house-dependent microflora variation (103, 150).

In a murine model of dextran sulfate sodium (DSS)-induced colitis, IL-1R8 deficiency is associated with increased local leukocyte infiltration and higher levels of proinflammatory cytokines (TNFα, IL- 6, IL- 1β, IL- 12p40, IL- 17), chemokines (CXCL1, CCL2), and prostaglandins, leading to an exacerbated intestinal inflammation. At the mechanistic level, this phenotype appears to be primarily due to the regulatory function of IL-1R8 in epithelial cells. These changes result in increased weight loss, intestinal bleeding, local tissue damage and reduced mice survival (103, 149), and susceptibility to Ulcerative Colitis-associated *E. coli* pathobionts (172). In addition, as reported above, IL-1R8 was essential for the anti-inflammatory role of IL-37 in this model *in vivo*, as well as in colonic organoids (173). In agreement with results obtained in mice, *SIGIRR* genetic variants and reduced expression of IL-1R8 as well as of IL-37 were shown to be associated with necrotizing enterocolitis in human (174, 175). In particular, a *SIGIRR* stop mutation (p.Y168X) was observed in an infant who died of severe necrotizing enterocolitis (174) and its functional effect was identified (176). The study showed that the p.Y168X mutation disrupted IL-1R8-mediated STAT3-dependent expression of miR-146a and miR-155, leading to deregulated IRAK1 activation and inflammation (176), thus identifying a new molecular mechanism underlying the regulatory role of IL-1R8.

In agreement with the concept that cancer-related inflammation contributes to cancer development and progression, IL-1R8 has been described to protect from cancer development in different murine tumor models. In a model that mimics intestinal cancer developed in Ulcerative Colitis patients, IL-1R8 deficiency was associated with increased intestinal inflammation and enhanced susceptibility to cancer development. IL-1R8 reduces the expression of NF-κB-induced genes critical for cell survival and proliferation (Bcl-xL and Cyclin D1), the local production of proinflammatory cytokines, chemokines and prostaglandin E_2,_ and intestinal permeability. Interestingly, the expression of IL-1R8 solely in gut epithelial cells rescues the phenotype of IL-1R8-deficient mice, reducing their susceptibility to colitis-associated cancer development and suggesting that the activity of IL-1R8 on tumorigenesis is mainly through its function on gut epithelial cells (149, 150).

In the genetic *Apc^min/+^* model, which resembles the Familial Adenomatous Polyposis syndrome (177), IL-1R8 deficiency led to increased tumorigenesis. IL-1R8-deficient mice show more sustained activation of the Akt/mTOR pathway in response to TLR or IL-1R ligands, leading to increased proliferation and chromosomal instability in cells of the colon crypts (114).

In human colorectal cancer, it has been shown that IL-1R8 expression is reduced compared with non-tumor tissues. Zhao et al. identified a dominant-negative isoform of IL-1R8 (IL-1R^δE8^), originated from a transcript lacking the exon 8, which is not modified by complex glycans and is retained in the cytoplasm. This isoform acts as a dominant-negative on IL-1R8, inhibiting its glycosylation, localization to the surface of colon epithelial cells and function. Indeed, in human colon cancer tissues IL-1R8 is cytoplasmic while in non-tumor tissue it has been found at the cell membrane. Importantly, transgenic mice expressing this mutant form of IL-1R8 in the colonic epithelium are more susceptible to colon cancer development in the colitis-associated tumor model, presenting higher local levels of inflammatory cytokines (IL-17A and IL-6) and the activation of transcription factors STAT3 and NF-κB. Taken together, these results suggest that complex glycan modifications and cell surface expression are required for IL-1R8 to reduce intestinal inflammation and tumorigenesis *in vivo* (178).

#### Breast Cancer

In breast cancer, the immunomodulatory role of IL-1R8 resulted in inhibition of IL-1-dependent NF-κB activation and production of pro-inflammatory cytokines *in vitro* and *in vivo*. In the genetic MMTVneu mouse model of breast cancer, IL-1R8-deficiency was associated with reduced mammary tumor growth and lung metastasis (151), protective tumor immune infiltrate characterized by higher frequency of DCs, NK cells and CD8^+^ T cells and reduced frequency of TAMs. According to these results, RNAseq analysis in 1102 clinical samples of breast tumors revealed that high IL-1R8 expression was associated with a non-T cell inflamed molecular signature, lower expression level of proinflammatory cytokines and chemokines, DC and NK cell metagenes, components of the peptide presenting machinery, cytolytic enzymes and type I interferon (IFN)-induced genes (151). Taken together these results suggest that IL-1R8 expression in breast tumors negatively regulates the mobilization and activation of immune cells and therefore promotes tumor growth and metastasis.

#### IL-1R8 as a Novel Checkpoint in NK Cells

Our group recently demonstrated that IL-1R8 is expressed by murine and human NK cells and its expression increases during NK cell differentiation. IL-1R8-deficiency was associated with increased frequency of mature NK cells in blood, spleen, bone marrow and liver. IL-1R8 deficiency resulted in enhanced expression of activating NK cell receptors and increased IFNγ, granzyme B, Fas ligand expression and degranulation. IL-1R8 directly acted on NK cells regulating responsiveness to IL-18, a key cytokine involved in NK cell activation, since the phenotype of IL-1R8 deficient mice was abrogated upon depletion of IL-18 or in IL-1R8/IL-18 double deficient mice. From a molecular point of view, IL-1R8 regulated IL-18-dependent activation of mTOR and JNK pathways. In agreement with these results, RNASeq and protein phosphorylation analysis showed that the response to IL-18 was affected in IL-1R8-deficient cells, in particular in the pathways involved in NK cell activation, degranulation, cytokine production and antiviral response. The relevance of these data was shown in models of hepatocellular carcinoma, sarcoma lung metastasis and colon cancer-derived liver metastasis, where IL-1R8 deficiency was associated with reduced liver disease severity, and lung and liver metastases. Further, in a model of murine cytomegalovirus (MCMV) infection, IL-1R8 deficient mice were more protected from the viral infection thank to enhanced NK cell degranulation and IFNγ production. Importantly, the adoptive transfer of IL-1R8 deficient NK cells was protective in the tumor metastasis and viral infection models. In human primary NK cells, IL-1R8 expression inversely correlated with IFNγ production, while IL-1R8 silencing resulted in increased IFNγ production and CD69 expression (94). Taken together, these results suggest that IL-1R8 blockade in NK cells, by tuning IL-18 signaling, may represent a novel therapeutic approach to unleash NK cell activity and strengthen NK cell antitumor and antiviral potential (**Table 1** and **Figure 4**).

#### Leukemia and Lymphoma

Deregulated TLR signaling has been associated with different B cell malignancies. In a mouse model of spontaneous chronic lymphocytic leukemia (CLL), IL-1R8 deficiency was associated with early onset of the monoclonal B cell expansion and reduced life span (152). In agreement, CLL cells expressed lower levels of IL-1R8 compare to B cells from healthy donors (101).

Chronic inflammation and in particular autoimmune disorders are linked with B-cell lymphoma development. In addition to inducing more severe autoimmunity in lpr mice (lupus prone strain), IL-1R8 deficiency increased the onset of DLBCL in aging mice due to a constitutive activation of NF-κB in splenocytes (153). Interestingly, IL-1R8 was downregulated in DLBCL compared to normal B cells, and its expression was positively associated with overall survival (153).

#### Contribution of IL-1R8 in the Antitumor Potential of IL-37

IL-37 has been shown to exert several inhibitory functions on tumor angiogenesis, migration and progression. As reported above, IL-37 interacts with IL-1R5/IL-18Rα and IL-1R8 to exert its anti-inflammatory activity (11). Further, IL-37 has been shown to compete with IL-18 for IL-1R5/IL-18Rα. For instance, in Oral Squamous Cell carcinoma (OSCC), IL-37 inhibited the proinflammatory effects of IL-18 and the increased IL-18/IL-37 ratio in serum predicted shorter overall and disease-free survival (179). In other contexts where IL-37 exerts an antitumor effect, it has not been proven yet whether it acts through IL-1R8. These include hepatocellular carcinoma, where IL-37 exerted an antiangiogenic role (180) or modulated the phenotype of TAMs by suppressing M2 polarization and regulating proinflammatory cytokine production (181), and Acute Myeloid Leukemia where IL-37 regulated IL-6 expression (182).

## Concluding Remarks

In the last 50 years, since the discovery of IL-1, the complexity of this cytokine family has been investigated and dissected, leading to the identification of a large number of molecules acting as accelerators and others acting as brakes (receptor antagonists, decoy receptors, negative regulatory receptors, anti-inflammatory ligands). This complexity underlines the relevance of fine tuning of IL-1 family-dependent functions both in homeostasis and disease. Further, unexpected functions for this family have emerged, demonstrating that its involvement is not restricted to infections and inflammation, but also impact degenerative conditions and cancer. Finally, the therapeutic potential of some members of the IL-1 system or, on the opposite, of their targeting, has been demonstrated in the last years, suggesting that fine dissection of their role, regulation and genetic variants may lead to the development of novel intervention strategies. In this general context, IL-1R8 and IL-1R2 emerge as tuners in various physiological and pathological conditions, which play essential functions to prevent immunopathology. On the other hand, their regulatory role may be exploited by escape mechanisms developed by pathogens and tumors, suggesting that their cell- and context-specific function must be dissected for the development of innovative therapeutic strategies.

## Author Contributions

All authors concurred in writing the manuscript. AM, LM, and DS prepared the figures. CG wrote and revised manuscript and figures. All authors contributed to the article and approved the submitted version.

## Conflict of Interest

The authors declare that the research was conducted in the absence of any commercial or financial relationships that could be construed as a potential conflict of interest.

## Publisher’s Note

All claims expressed in this article are solely those of the authors and do not necessarily represent those of their affiliated organizations, or those of the publisher, the editors and the reviewers. Any product that may be evaluated in this article, or claim that may be made by its manufacturer, is not guaranteed or endorsed by the publisher.
